# Personalizing Care for Critically Ill Adults Using Omics: A Concise Review of Potential Clinical Applications

**DOI:** 10.3390/cells12040541

**Published:** 2023-02-08

**Authors:** Kay Choong See

**Affiliations:** Division of Respiratory and Critical Care Medicine, Department of Medicine, National University Hospital, Singapore 119228, Singapore; kaychoongsee@nus.edu.sg

**Keywords:** acute kidney injury, critical illness, genomics, metabolomics, precision medicine, proteomics, respiratory distress syndrome, sepsis

## Abstract

Current guidelines for critically ill patients use broad recommendations to promote uniform protocols for the management of conditions such as acute kidney injury, acute respiratory distress syndrome, and sepsis. Although these guidelines have enabled the substantial improvement of care, mortality for critical illness remains high. Further outcome improvement may require personalizing care for critically ill patients, which involves tailoring management strategies for different patients. However, the current understanding of disease heterogeneity is limited. For critically ill patients, genomics, transcriptomics, proteomics, and metabolomics have illuminated such heterogeneity and unveiled novel biomarkers, giving clinicians new means of diagnosis, prognosis, and monitoring. With further engineering and economic development, omics would then be more accessible and affordable for frontline clinicians. As the knowledge of pathophysiological pathways mature, targeted treatments can then be developed, validated, replicated, and translated into clinical practice.

## 1. Introduction

Current guidelines for critically ill patients use broad recommendations to promote uniform protocols for the management of conditions such as acute kidney injury (AKI), acute respiratory distress syndrome (ARDS), and sepsis [1,2,3]. Although these guidelines have enabled the substantial improvement of care, mortality for critical illness remains high. One reason for this may be that a one-size-fits-all method may not be the optimal approach and does not work for all patients with the same diagnostic label. Even for the same disease, critically ill patients respond differently and have variable disease trajectories and outcomes. In addition, critically ill patients derive differential benefits from the same treatment. Therapeutic trials on unselected patients sometimes fail to demonstrate any benefit while trials for carefully selected patients succeed in doing so (e.g., steroid therapy for septic shock [4]). Further outcome improvement would therefore involve understanding the sources of disease heterogeneity and tailoring management strategies for different patients.

However, the accurate determination of disease heterogeneity based only on routinely collected clinical data has not been always possible. Genomic, transcriptomic, metabolomic, and proteomic technologies have therefore been developed to allow the identification and quantitative analysis of components from biological samples (e.g., blood, urine, respiratory secretions, tissue). These components may help to differentiate between different types of disease-related damage and predict clinical risk and outcomes. Better knowledge of pathophysiological pathways of disease offers opportunities for the development of diagnostic tools and personalized therapy for various critical illnesses. Better prognostic tools can help identify high risk individuals who might benefit from more aggressive or pre-emptive interventions.

Omics have been clinically applied to personalize care in several fields of medicine, improving diagnosis, prognosis, and treatment. In cardiology, gene expression profiling tests carried out in heart transplant endomyocardial biopsies can help identify heart transplant recipients’ probability of antibody-mediated rejection and heart allograft loss [5]. In oncology, tumor tissue or blood-based genomics have been used to guide targeted therapy for cancer [6]. Referencing published studies in humans as examples, this review will focus on potential clinical applications using omics to personalize care for adults with the following major forms of critical illness: AKI, ARDS, and sepsis (both COVID-19- and non-COVID-19-related).

## 2. Types of Omics

Four broad areas define the cellular and molecular mechanisms of disease: genomics, transcriptomics, proteomics, and metabolomics. Genomics refers to the study of genes, genetic variants, and gene interactions. In the context of infection, genomic analysis may be performed for both host and microbes within a sample (e.g., blood), and the latter is called metagenomics. Transcriptomics refers to the study of ribonucleic acid molecules within a sample, providing a link between genomics and proteomics. Proteomics refers to the proteins translated in an organism. Metabolomics refers to the small molecules and metabolites identified within a biological sample. The term metabolomics includes lipidomics, which is the large-scale study of the pathways and networks of cellular lipids in biological systems.

The transcriptome includes protein-coding and non-protein-coding RNAs. Transcription within cells produces ribonucleic acids (RNAs) that are based on the genomic template, which encodes more than 20,000 different RNAs. In turn, messenger RNAs (mRNA) code for proteins, while other types of RNA are noncoding. Together with mRNA, ribosomal RNA (rRNA) and transfer RNA (tRNA) are involved in protein assembly. Other types of RNA are involved in various cellular processes and have tissue-specific patterns of expression that are specific and time-dependent. For instance, micro-RNAs (miRNAs) are 21–24 nucleotide-long duplex noncoding RNAs that regulate gene expression by binding to complementary sequences on mRNA. Such binding prevents the translation of mRNA sequences into protein, and each miRNA may regulate hundreds of targets. Circulating miRNAs are particularly suitable for analysis as they are protected against degradation by inclusion in extracellular micro-vesicles or by the formation of protein-miRNA complexes.

Post-transcriptional regulation and degradation would alter protein expression. In the field of proteomics, proteins that are translated in cells and tissues at specific times can be analyzed and may be subsequently tapped as diagnostic and prognostic markers. Further downstream, cellular processes give rise to an array of metabolites, which can be similarly analyzed (metabolomics) to provide an integrated understanding of upstream genomic, transcriptomic, and proteomic processes.

Technology used to uncover disease-associated genes, ribonucleic acid molecules, proteins, and metabolites differ. The identification of disease-associated genes can be carried out using a candidate gene approach, micro-array analysis, genome-wide association study (GWAS) [7], whole-genome sequencing (WGS), and whole-exome sequencing (WES). Transcriptomic technologies involve microarrays (which quantify known sequences) and high-throughput sequencing (which is carried out by reverse transcribing RNA and synthesizing complementary DNA, which can then be sequenced).

Screening for differentially expressed proteins requires liquid chromatography–mass spectrometry (LC-MS) (for liquid samples) and matrix-assisted laser desorption/ionization time of flight (MALDI-TOF) mass spectrometry, which may be applied to various types of body fluids and tissue samples. After screening, selected differentially expressed proteins can then be quantified by enzyme-linked immunosorbent assays (ELISA). Separately, the assay of metabolites is performed using LC-MS (for liquid samples), gas chromatography–mass spectrometry (GC-MS) (for gaseous samples), or nuclear magnetic resonance (NMR) spectroscopy [8,9].

## 3. Omics for Acute Kidney Injury

### 3.1. Definition and Current Management of Acute Kidney Injury

AKI is defined as an abrupt reduction of glomerular filtration rate over a period of one week or less and increases the risk of long-term dialysis dependence and mortality (~23.9%) [1,10]. The diagnosis of AKI requires creatinine elevation or decreased urine output [11]. Serum creatinine elevation is relatively insensitive to kidney injury and requires knowledge of “baseline” creatinine. Urine output is a more sensitive marker of kidney injury than serum creatinine but remains imperfect as non-oliguric AKI may occur.

The current management of AKI requires a thorough physical examination, which may be augmented by point-of-care ultrasound to reveal a specific etiology, e.g., hypovolemia (presence of small cardiac chambers) or post-renal obstruction (presence of dilated renal calyces suggesting hydronephrosis or a distended post-void bladder suggesting bladder outlet obstruction) [12]. While reversing specific causes, patients require the avoidance of nephrotoxins. The progression of AKI necessitates therapeutic escalation including kidney replacement therapy (KRT) (also known as renal replacement therapy or RRT) when life-threatening conditions are present or when serum urea exceeds 40 mmol/L [13].

### 3.2. Application of Omics for Acute Kidney Injury

Beyond the standardized and protocolized management of AKI for critically ill patients, several studies illustrate the potential for improved diagnosis with proteomics and more accurate prognostication with genomics and transcriptomics (Table 1). These applications pivot around two main mechanisms. As renal tubular cell death and tubulointerstitial inflammation are the main determinants of tissue damage and kidney failure, omics studies unsurprisingly reveal pathways leading to apoptosis [14,15,16,17] and inflammation [18,19,20].

#### 3.2.1. Omics Studies Involving Apoptosis Pathways

Li and colleagues performed a combined animal and human study to discover new biomarkers for early AKI diagnosis [14]. Urine samples were collected from septic rats and analyzed using LC-MS. Using the differential expression of urinary proteins in rats, proteins PARK7 and CDH16 were found to be correlated (positively and negatively, respectively) with serum creatinine elevation among 30 septic humans with AKI and 59 humans without AKI. PARK7 is known to upregulate androgen receptor-dependent transcription, inhibit cellular oxidative stress, and regulate mitochondrial function, autophagy, and apoptosis. CDH16 is a calcium-dependent, membrane-associated glycoprotein that is exclusively expressed in the kidney, where it plays a role in the morphogenic direction of tissue development. Receiver Operating Characteristic (ROC) curve analysis revealed that PARK7 and CDH16 could discriminate AKI versus no AKI with the area under the receiver operating characteristic curve (AUC) of 0.900 and 0.898. The cutoff values of PARK7 and CDH16 for identifying AKI were 13.557 ng/mL (sensitivity 86.7%, specificity 96.6%) and 53.29 pg/mL (sensitivity 83.3%, specificity 94.9%), respectively.

Frank and colleagues studied 887 US patients with septic shock, aiming to uncover genetic predictors for AKI [15], performing genotyping using the Human-CVD BeadChip (Illumina, San Diego, CA, USA), a multisample genotyping panel including 48,742 markers across approximately 2100 genes. They then discovered five single nucleotide polymorphisms (SNP) in apoptosis-related genes *BCL2*, *SERPINA4*, *SERPINA5*, and *SIK3*. Two of these SNPs were in the *BCL2* gene and were protective against AKI: rs8094315 (odds ratio [OR] 0.61 per additional copy of the minor G allele, *p* = 0.016) and rs12457893 (OR 0.71 per additional copy of the minor C allele, *p* = 0.026 in validation set). One SNP (rs625145) was in the *SIK3* gene and was associated with an increased risk of AKI (OR 1.64 per additional copy of the minor T allele, *p* = 0.029 in the validation set). The final two SNPs were rs2093266 (in the *SERPINA4* gene, OR 0.55, *p* = 0.031) and rs1955656 (in the *SERPINA5* gene and in complete linkage disequilibrium with rs2093266). Vilander and colleagues attempted to validate the findings by Frank and colleagues in a separate Finnish cohort of 2567 critically ill patients with sepsis/septic shock but without chronic kidney disease [16]. However, they found that *SERPINA4* and *SERPINA5*, but not *BCL2* and *SIK3*, were associated with AKI.

Sun and colleagues were interested in studying single nucleotide polymorphisms (SNPs) (rs41275743 and rs4648143) in the 3′-untranslated region of the nuclear factor-kappaB gene *NFKB1* and the risk of acute kidney injury (AKI) in sepsis [17]. Nuclear factor-kappaB is a widely expressed animal protein complex that controls DNA transcription, cytokine production, and cell survival. Among 235 AKI patients and 235 non-AKI patients, they found that SNP rs41275743 raised the risk of AKI by 1.46 times, while SNP rs4648143 raised the risk of AKI by 1.56 times, independent of the Acute Physiology and Chronic Health Evaluation (APACHE) III score, Simplified Acute Physiological Score (SAPS) II, and Sequential Organ Failure Assessment (SOFA) score.

#### 3.2.2. Omics Studies Involving Inflammatory Pathways

Genga and colleagues examined 202 patients with sepsis from a Canadian Emergency Department cohort and validated their findings in 604 patients from the Vasopressin in Septic Shock Trial (VASST) [18]. The investigators found that one genetic variant in the Cholesteryl Ester Transfer Protein (CETP) gene, rs1800777 (allele A), was strongly associated with lower high-density lipoprotein cholesterol (HDL-C) levels and increased risk of AKI (OR 2.38, *p* = 0.020). A plausible explanation was that the genetic variant was also associated with cumulative fluid balance and pro-inflammatory cytokines. This association was supported by Mendelian randomization analysis, which raised the possibility that CETP inhibition could prevent or treat AKI.

Given that disturbances in iron metabolism are associated with inflammation and that oxidative stress is involved in AKI pathogenesis, Vilander and colleagues performed the targeted genotyping of 300 patients with severe AKI (KDIGO 2 or 3) and 353 controls without AKI (KDIGO 0) for the guanine-thymine (GTn) repeat in the promoter region of the heme oxygenase-1 gene (*HMOX1*) [19]. Similarly to previous investigators [20], they found an association between a short repeat in *HMOX1* and AKI risk (OR 1.30 for each S-allele in an additive genetic model, 95% CI 1.01–1.66, *p* = 0.041). Alleles with a repeat number greater than 34 were significantly associated with lower cytoprotective heme oxygenase-1 (HO-1) concentration (*p* < 0.001).

#### 3.2.3. Omics Studies Involving Multiple Pathways

Zhang and colleagues studied 172 patients with sepsis participating in the Chinese Multi-omics Advances in Sepsis (CMAISE) and measured gene expression in peripheral blood mononuclear cells (PBMC) taken on the first day of admission. Using RNA sequencing, the investigators identified and developed a 43-gene signature support vector machine (SVM) model, which was significantly better at predicting persistent AKI than comprehensive 58-variable clinical model based on demographics, comorbid conditions, vital signs, treatments administered, and laboratory findings (AUC 0.948, 95% CI 0.912–0.984 versus 0.739, 0.648–0.830, *p* < 0.01) [21].

## 4. Omics for Acute Respiratory Distress Syndrome

### 4.1. Definition and Current Management of Acute Respiratory Distress Syndrome

ARDS is severe lung failure with an incidence of 3.65–58.7 per 100,000 person-years for moderate-to-severe ARDS and a 90-day mortality rate of 38–47% in population-based studies [22]. The pathophysiology of ARDS involves neutrophil accumulation and activation in the lungs, cellular release of inflammatory mediators and cytokines, and eventual diffuse alveolar damage. The clinical diagnosis of ARDS requires a combination of four features: (1) acute hypoxemic respiratory failure; (2) onset of lung injury within one week of a known clinical insult or new or worsening respiratory symptoms; (3) bilateral radiographic opacities; and (4) respiratory failure not fully explained by cardiac failure or fluid overload [23]. In addition, ARDS can only be diagnosed under the conditions of positive pressure ventilation with a minimum positive end-expiratory pressure (PEEP) of 5 cm H_2_O delivered either non-invasively or invasively. Treatments for ARDS are generally supportive, such as low tidal volume ventilation (4–6 mL/kg of predicted body weight), controlled plateau pressure (<30 cm H_2_O), low driving pressure (<15 cm H_2_O), adequate PEEP according to the PEEP-FIO2 table (FIO2 being the inspired oxygen fraction) [24], neuromuscular blocking agents, prone positioning, recruitment maneuvers, and extracorporeal membrane oxygenation [3].

ARDS classification and phenotyping have been carried out using ventilatory variables (e.g., P/F ratio (P/F being the ratio of arterial oxygen partial pressure and FIO2), dead space fraction, PEEP, ventilatory ratio, driving pressure) and biological features (e.g., focal versus diffuse distribution of pulmonary infiltrates [25], inflammatory and coagulative biomarkers [26]). Of these, the P/F ratio has played a central role for prognostication, risk stratification, and guidance for therapy. However, the P/F ratio and other means of phenotyping are not specific to ARDS. They seem to reflect overall severity and the need for escalation of respiratory support, rather than differences in the pathophysiological mechanism. Even attempts at distinguishing pulmonary from extrapulmonary triggers for ARDS do not work well, as different sites of primary disease have not been demonstrated to operate via different pathophysiological pathways towards lung injury.

### 4.2. Application of Omics for Acute Respiratory Distress Syndrome

Given the limited ability of current clinical methods to uncover sufficient disease heterogeneity for guiding personalized care, the following omics-based studies provide promise of future improvement (Table 2). In these examples, proteomic and transcriptomic signatures may enhance diagnostic accuracy or provide a means for early diagnosis of ARDS. Transcriptomics, proteomics, and metabolomics may improve prognostication. Finally, metabolomic profile changes over time can help monitor a patient’s trajectory. Reflecting a high degree of heterogeneity within the ARDS clinical syndrome, omics studies demonstrate mechanistic pathways that generally fall into four disparate groups: apoptosis/repair [27,28], inflammation [29,30,31,32,33,34,35,36,37], pulmonary vascular injury [38,39], and energy metabolism [9].

#### 4.2.1. Omics Studies Involving Apoptosis Pathways

Rahmel and colleagues compared levels of miRNA 122 in blood samples from 119 acute respiratory distress syndrome (ARDS) patients within the first 24 h of intensive care unit (ICU) admission and 20 patients undergoing elective abdominal non-liver surgery [27]. Of the 119 patients with ARDS, 16 patients (13%) developed ALF and 37 patients (31%) died at 30 days of follow-up. miRNA 122 is a liver-specific miRNA which comprises 70% of all liver miRNAs and is minimally expressed in other tissues. It controls hepatocyte differentiation, proliferation, and apoptosis. When hepatocyte damage occurs, blood miRNA 122 increases earlier than blood alanine aminotransferase [40]. In line with the known association of miRNA 122 with liver disease, miRNA expression was elevated early in patients who developed acute liver injury (defined as total bilirubin concentration ≥3.0 mg/dl, alanine transferase ≥350 U/L, and international normalized ratio ≥2.0) and in non-survivors at 30 days.

Molinero and colleagues studied bronchial aspirates from 74 critically ill (14 non-COVID-19, 60 COVID-19) patients with COVID-19 and non-COVID-19 ARDS [28]. A total of 64 proteins were quantified using targeted proximity extension assay. In COVID-19 patients, reduced levels of ENTPD2 and PTN (a multifunctional trophic factor that participates in cell differentiation, proliferation, and growth) were observed in patients who died in ICU, suggesting that inappropriate repair was linked to fatal outcomes. Separately, a protein-based model using AGR2, NQO2, IL-1α, OSM, and TRAIL was constructed to predict in-ICU mortality. Furthermore, among 23 ARDS survivors, the levels of proteins FCRL1, NTF4, and THOP1 correlated with the diffusing capacity for carbon monoxide (diffusing capacity of the lung) (DLCO) 3 months after hospital discharge.

#### 4.2.2. Omics Studies Involving Inflammatory Pathways

Using LC-MS, Ren and colleagues performed screening proteomic analysis on plasma and bronchoalveolar lavage samples from 37 patients with pneumonia (5 without ARDS, 16 with mild ARDS, and 16 with severe ARDS, according to the Berlin criteria), and quantified selected differentially expressed proteins in plasma with ELISA [29]. They found that plasma malignant brain tumors 1 protein (DMBT1) was elevated in early ARDS compared to no ARDS (mean plasma concentration 2160.50 ± 94.06 vs. 1752.60 ± 111.68 pg/mL, *p* < 0.05) and was higher in severe ARDS compared to mild ARDS (mean plasma concentration 2255.26 ± 143.00 vs. 2160.50 ± 94.06 pg/mL, *p* < 0.05). DMBT1 protein functions in innate immunity, inflammation, and epithelial cell differentiation. As such, DMBT1 could serve as a biomarker both for diagnosis and severity assessment in ARDS.

Bos and colleagues analyzed the exhaled breath from 101 ventilated intensive care unit patients using GC-MS on the first day of admission [30]. A total of 42 of these patients had ARDS, as defined by the Berlin criteria, 3 had pneumonia, 4 had cardiogenic pulmonary edema, and 52 had other medical/surgical conditions. Three breath metabolites, octane, acetaldehyde, and 3-methylheptane, were able to discriminate between ARDS and controls with an AUC of 0.80. Octane and 3-methylheptane are produced by lipid peroxidation during oxidative stress, while acetaldehyde is produced by leukocytes, which infiltrate the lungs in ARDS. The combination of the three-metabolite panel with the lung injury prediction score increased the AUC to 0.91.

Goodwin and colleagues collected plasma from intensive care unit patients with a primary diagnosis of sepsis [31]. The higher circulating level of miRNA 887 was associated with sepsis-associated ARDS development. Additionally, the transfection of miRNA 887 into human pulmonary microvascular endothelial cells (HPMECs) upregulated several genes previously associated with ARDS (*CXCL10*, *CCL5*, *CX3CL1*, *VCAM1*, *CASP1*, *IL1B*, *IFNB*, and *TLR2*) and activated cellular pathways that increased endothelial chemokine release and trans-endothelial leukocyte migration.

Shi and colleagues analyzed data from the Gene Expression Omnibus databases in the National Center for Biotechnology Information, which used a transcriptomic approach to uncover the gene *ADORA3* as a predictor of prolonged time on mechanical ventilation and mortality at 28 days [32]. The unbiased genome-wide transcriptional profiling of alveolar macrophages purified from 68 bronchoalveolar lavage samples from 35 patients with ARDS allowed the investigators to link *ADORA3* with significantly augmented sphingolipid signaling and cGMP-PKG signaling pathways and neuroactive ligand-receptor interaction. These pathways induce pro-inflammatory cytokines, immune-mediated damage, and fibrosis.

Using high-throughput sequencing, Zhang and colleagues compared serum samples from 20 healthy adult controls and 21 adult patients with ARDS and found two miRNAs (miRNA 584 and miRNA 146a) that were significantly downregulated in the latter [33]. The target transcripts of these two miRNAs were implicated in the regulation of various inflammatory factors, with the transcription factor nuclear factor kappa B (NF-kappaB) playing an important role in this process. miRNA 584, miRNA 146a, and NF-kappaB may therefore all be promising therapeutic targets for patients with ARDS.

Zhu and colleagues performed the Molecular Epidemiology Study of ARDS (MEARDS) at the Massachusetts General Hospital and Beth Israel Deaconess Medical Center intensive care units, using the TaqMan OpenArray Human miRNA Panel (Applied Biosystems, Foster City, CA, USA) for miRNA profiling. From 78 whole blood samples, 754 miRNAs were identified [34]. Using differential expression and backwards elimination, eight miRNAs were combined into a transcriptomic classifier that predicted increased 28-day mortality in moderate-to-severe ARDS. The eight miRNAs were miRNA 628.3p, miRNA 922, miRNA 505, miRNA 130b, miRNA 624, miRNA 766, miRNA 194, and miRNA 7, with individual hazard ratios for 28-day mortality ranging from 1.05–1.70, adjusted for age, gender, sepsis, pneumonia, and APACHE III score.

Dong and colleagues studied 300 patients with moderate to severe ARDS, with 99 patients (33%) dying within 28 days [35]. Using immunoassay, they found that plasma Insulin-like Growth Factor Binding Protein 7 (IGFBP7) moderately increased ARDS 28-day mortality (OR 1.11, 95% CI 1.04–1.19, *p* = 0.002) per log_2_ increase (i.e., per two-fold increase). Causal mediation analysis indicated that the association between IGFBP7 and ARDS 28-day mortality was mediated by platelet count (OR 1.03, 95% CI 1.02–1.04, *p* = 0.01), which was consistent with platelet involvement in ARDS-associated inflammation and disseminated intravascular coagulation.

Liu and colleagues studied 51 patients with severe ARDS receiving extracorporeal membrane oxygenation (ECMO) and examined whether the plasma levels of interleukin-10 (a pro-inflammatory cytokine) predicted outcomes [36]. A total of 30 patients died in ICU, 1 patient died outside of the ICU, and 20 survived to hospital discharge. Using enzyme-linked immunosorbent assay (ELISA), the overproduction of interleukin-10 was correlated with the development of renal failure and eventual hospital mortality, independent of age, etiology of ARDS, immunocompetence, body mass index, and the duration of mechanical ventilation before ECMO initiation. A threshold interleukin-10 concentration of 88.9 pg/mL predicted ICU mortality with a sensitivity of 73.3% and a specificity of 90.5%, comparable to the SOFA and APACHE II scores.

Xu and colleagues studied the metabolomic profiles of blood plasma from 42 ARDS patients and 28 healthy controls using LC-MS [37]. The levels of phenylalanine, D-phenylalanine, and phenylacetylglutamine were significantly increased in non-survivors compared to the survivors of ARDS. For predicting mortality, the respective AUCs for phenylalanine, D-phenylalanine, and phenylacetylglutamine were 0.803, 0.785, and 0.709, respectively. Additionally, the injection of phenylalanine into an ARDS mouse model increased lung injury and mortality, and investigators hypothesized a pro-inflammatory role for phenylalanine.

#### 4.2.3. Omics Studies Involving Pulmonary Vascular Injury Pathways

As part of the Molecular Epidemiology Study of ARDS (MEARDS), among 530 patients with critical ARDS and non-ARDS illnesses, the whole blood screening of 294 miRNA candidates identified three miRNAs that could be used to predict ARDS: miRNA 181a and miRNA 92a were risk biomarkers for ARDS, whereas miRNA 424 was a protective biomarker [39]. These findings are consistent with the known roles of miRNA 181a (regulates airway inflammation and neutrophils), miRNA 92a (inhibits endothelial cell angiogenesis, impairs endothelial cell function, and promotes inflammatory responses), and miRNA 424 (activated in endothelial cells to stabilize hypoxia inducible factors, which may counter pulmonary vasoconstriction, inflammation, and tissue damage). The addition of these miRNA biomarkers to the Lung Injury Prediction Score increased discrimination for ARDS development, with the AUC increasing from 0.708 to 0.723, with a category-free net reclassification index of 27.21% (*p* = 0.01).

Price and colleagues used targeted proteomics, employing the Olink proximity extension assay (Uppsala, Sweden), to identify an interesting low vascular injury signature, which included 22 proteins associated with vascular injury, platelet levels, and vascular function [38]. This signature of low vascular protein abundance was significantly associated with lower platelet count and higher mortality in 60 ARDS patients.

#### 4.2.4. Omics Studies Involving Energy Metabolism

Yan and colleagues performed nuclear magnetic resonance-based metabolomics analyses of serum and urine samples, before and after the treatment of community-acquired pneumonia with ARDS (43 patients) and without ARDS (45 patients) [9]. A total of 20 serum metabolites were identified, and these were mainly involved in energy, lipid, and amino acid metabolism. A total of 42 urinary metabolites were identified, and these were mainly involved in energy metabolism. Elevated levels of serum 3-hydroxybutyrate, lactate, acetone, and acetoacetate and decreased levels of serum leucine, choline, and urine creatine and creatinine were detected in patients with community-acquired pneumonia and ARDS versus those without ARDS. Serum metabolites 3-hydroxybutyrate, acetone, acetoacetate, citrate, and choline were able to predict the improvement of community-acquired pneumonia and ARDS with an AUC of 0.866. Urinary metabolite 1-methylnicotinamide was able to predict the improvement of community-acquired pneumonia and ARDS with an AUC of 0.795. A combination of serum and urine metabolites was able to predict the improvement of community-acquired pneumonia and ARDS with an AUC of 0.952.

## 5. Omics for Sepsis

### 5.1. Definition and Current Management of Sepsis

Sepsis is a life-threatening condition caused by a dysregulated response to infection, which may in turn lead to multiple organ failure [2]. Sepsis is a common cause of death globally [41]. Improved survival rests on the rapid administration of antibiotics and fluids, with early appropriate blood culture [42].

Despite the prompt and appropriate administration of antimicrobials and fluid therapy, mortality remains high in sepsis. One reason could be the late recognition of sepsis, given nonspecific symptoms and signs, especially in older patients [43]. Another reason could be that microbial culture is relatively slow and has poor sensitivity. As every hour of delayed antibiotic administration increases the risk of death from septic shock by 7.6% [44], the lack of an early culture result compels the use of empiric broad spectrum antimicrobials. Empiric antimicrobial therapy may however miss the mark in two ways [45] and increase the risk of mortality: firstly, when it does not cover for the correct pathogen; secondly, when the pathogen has developed resistance to the antimicrobial.

### 5.2. Application of Omics for Sepsis

Several examples demonstrate the clinical potential of omics to improve diagnosis, prognosis, and treatment of sepsis (Table 3). Given the known association of sepsis with systemic inflammatory response, microcirculatory dysfunction, and endothelial dysfunction, most omics studies are unsurprisingly linked to inflammatory pathways [28,46,47,48,49], microcirculatory function [50], and endothelial function [7,51]. Some of these examples also demonstrate the important role of metagenomics [49,52]. Genetic material recovered from bacteria, fungi, and viruses can direct specific antimicrobial therapy in the context of infection and can provide early guidance in the absence of culture results.

#### 5.2.1. Omics Studies Involving Inflammatory Pathways

Kapp and colleagues studied 107 patients who developed sepsis from primary intra-abdominal infections [28]. They employed discovery-based plasma proteomics using LC-MS and identified 49 proteins whose expression levels were associated with both survival outcome and racial background. These proteins were involved in the acute phase response and complement system, which are key inflammatory pathways related to sepsis. A smaller set of 19 proteins, involved in liver cell activation, were significantly represented in patients regardless of racial/ethnic background and may represent potential universal molecular changes in sepsis.

Nguyen and colleagues studied 30 patients with acute lung injury (mild ARDS), 14 of whom subsequently developed ventilator-associated pneumonia (VAP) [46]. The investigators performed exploratory proteomic analysis on bronchoalveolar lavage fluid using LC-MS and found that three proteins (S100A8, lactotransferrin, actinin 1) discriminated between patients with and without ventilator-associated pneumonia. The investigators hypothesize that S100A8 recruits neutrophils to sites of inflammation in response to lipopolysaccharide, while lactotransferrin is elevated as part of the respiratory tract antimicrobial defense system and innate immunity. This three-protein signature can be more quickly analyzed than standard microbiological tests and had an optimal sensitivity–specificity profile of 93% and 94%, respectively. Proteomics may help improve both the speed and accuracy of VAP diagnosis, given that the clinical features for VAP are nonspecific.

Wang and colleagues analyzed the plasma samples of 196 sepsis patients and 196 healthy controls [47]. Using quantitative reverse transcription polymerase chain reaction, the investigators detected lower levels of two microRNAs (miRNA 103 and miRNA 107) in sepsis patients compared to healthy controls. These two microRNAs were inversely associated with APACHE II score, SOFA score, serum creatinine, C-reactive protein, tumor necrosis factor, interleukin 1β, interleukin 6, and interleukin 8. In addition, decreased miRNA 103 and miRNA 107 both predicted a risk of concomitant acute respiratory distress syndrome and 28-day mortality. Their findings are consistent with the known roles of miRNA 103 and miRNA 107 as the regulators of several inflammation-related pathways involving mitogen-activated protein kinase (MAPK), extracellular regulated protein kinases (ERK), Notch signaling, and the NF-kappaB.

Ruiz-Sanmartín studied 141 patients with sepsis and used LC-MS to identify 177 proteins [48]. Using recursive feature elimination classification and cross-validation with a vector classifier, they found that nine proteins (GPX3, APOB, ORM1, SERPINF1, LYZ, C8A, CD14, APOC3, and C1QC) were associated with organ dysfunction (defined by SOFA score > 6) with a sensitivity of 81%, a specificity of 84%, and an of AUC 0.82. In addition, they found that 22 proteins (CLU, LUM, APOL1, SAA1, CLEBC3B, C8A, ITIH4, KNG1, AGT, C7, SAA2, APOH, HRG, AFM, APOE, APOC1, C1S, SERPINC1, IGFALS, KLKB1, CFB, and BTD) were associated with mortality with a sensitivity of 91%, a specificity of 72%, and an AUC of 0.81. Some of these proteins are known to be involved in inflammation (APOC1, APOC3, KNG1), proteolysis (SERPINF1, CLEC3B), and immunity (C1S, C8A).

Peng Wu and colleagues collected blood samples from 231 COVID-19 patients with varying disease severity, ranging from asymptomatic to critically ill [49]. Whole genome sequencing was used to obtain genomic data, RNA sequencing for transcriptomic data, and liquid chromatography–mass spectrometry for proteomic/metabolomic data. This “trans-omics” approach encompassing genomic, transcriptomic, proteomic, and metabolomic profiles revealed neutrophil heterogeneity between asymptomatic and critically ill patients. Asymptomatic COVID-19 patients had high neutrophil counts and enhanced IFN antiviral response compared to more severely ill patients. In contrast, critically ill COVID-19 patients, neutrophil over-activation, cytokine storm, and IFN-mediated innate immunity or T-mediated adaptive immunity deficiency were present. In addition, in critically ill patients, arginine depletion and tryptophan metabolites accumulation occurred, which correlated with T cell dysfunction.

#### 5.2.2. Omics Studies Involving Microcirculatory Function

Rovas and colleagues studied 22 adult COVID-19 patients, 43 bacterial sepsis patients, and 10 healthy controls from four hospitals [50]. All patients underwent sublingual microscopy to measure microcirculatory parameters and targeted proteomics using the Olink proximity extension assay (Uppsala, Sweden). The investigators found two clusters of proteins and consistent results between bacterial sepsis and COVID-19. Cluster 1 contained a total of 23 unique proteins, including von Willebrand factor-cleaving protease (ADAMTS13), Angpt-1, and VEGF-D; this cluster was positively correlated with microvascular health and negatively correlated with microvascular injury. Cluster 2 contained 100 unique proteins, including IL2, IL6, IL8, IL10, IL14, IL16, and various pro-inflammatory mediators; this cluster was negatively associated with microvascular health. The ratio of Cluster 1 and Cluster 2 was used as a proteomic signature of microvascular dysfunction, which predicted the composite endpoint of 28-day mortality and/or intubation with an AUC of 0.90 (95% CI 0.86–0.94, *p* < 0.0001).

#### 5.2.3. Omics Studies Involving Endothelial Function

Li and colleagues used LC-MS to study the serum proteomic profiles of 53 non-septic patients, 37 sepsis patients, and 35 septic shock patients [51]. An index combining the levels of four proteins (β2-microglobulin >3.7 mg/L, VCAM1 >2216.8 ng/mL, ApoC3 ≤54.532 μg/mL, ApoE >62.45 mg/L) and two conventional infection biomarkers (procalcitonin, C-reactive protein yielded an AUC of 0.772 for sepsis identification. The investigators hypothesized that VCAM1 reflected endothelium dysfunction, β2-microglobulin reflected sepsis-induced kidney, ApOC3 reflected hepatic secretory function, and ApoE reflected coagulation system failure.

Rautanen and colleagues carried out a GWAS in 2534 white adult patients with sepsis due to pneumonia who had been admitted to ICUs [7]. They found that common variants in the *FER* (Fps/Fes related tyrosine kinase) gene were strongly associated with survival, with each allele reducing 28-day mortality by 44% (hazard ratio for death 0.56, 95% CI 0.45–0.69). Mortality was 9.5% in patients carrying the CC genotype, 15.2% in those carrying the TC genotype, and 25.3% in those carrying the TT genotype (wild type homozygous). The *FER* gene is widely expressed in tissues and encodes a cytosolic non-receptor tyrosine kinase which influences host defense against infection, neutrophil chemotaxis, and endothelial permeability. Apart from being a biomarker for risk stratification, the *FER* gene and associated molecular pathways are potential novel targets for sepsis prevention or treatment.

#### 5.2.4. Omics Studies Involving Metagenomics

Chien and colleagues compared the metagenomic next-generation sequencing of microbial DNA/RNA versus culture (bacterial, fungal, viral) in 50 patients [52]. When the comparison was carried out using blood samples, next-generation sequencing had higher detection rates than blood culture (88.0% vs. 26.0%, *p* < 0.001). When the comparison was performed using bronchoalveolar fluid samples, next-generation sequencing had higher detection rates than lavage culture (92.0% vs. 76.0%, *p* = 0.054).

Although the detection rates using omics were higher than conventional culture, one possible limitation—which also affects conventional cultures—is to distinguish pathogenic microbes from colonizers/contaminants when infection is uncertain. Kalantar and colleagues provide a possible means to overcome this limitation by carrying out the integrated host and pathogen metagenomic RNA and DNA next generation sequencing (mNGS) of whole blood and plasma from 321 critically ill patients. The investigators aimed to use whole-blood transcriptional profiling to distinguish infectious from non-infectious conditions and viral from bacterial infections [49]. Patients deemed to have an infection could then undergo broad-range pathogen identification and pathogen-specific treatment. For sepsis diagnosis, plasma RNA yielded a transcriptional signature with an AUC of  0.77 for the validation set. For diagnosing a viral pathogen as the cause of sepsis, a secondary transcriptomic classifier had an AUC of 0.96 for the validation set. An integrated sepsis diagnostic model then identified 99% of microbiologically confirmed sepsis cases and predicted sepsis in 74% of suspected and 89% of indeterminate sepsis cases.

#### 5.2.5. Omics Studies Involving Multiple Pathways

Su and colleagues compared plasma and urine metabolomic profiles of 30 septic patients and 35 non-septic ICU controls using LC-MS [53]. Ten amino acid and lipid metabolites (PE (20:4(5Z, 8Z, 11Z, 14Z)/P-18:0), harderoporphyrinogen, chloropanaxydiol, (Z)-2-octenal, N1,N8-diacetylspermidine, 1-nitroheptane, venoterpine, α-CEHC, LysoPE (20:0/0:0) and corticrocin were indicators for sepsis. A regression model incorporating these ten metabolic biomarkers and five traditional physiological indicators had a perfect AUC of 1.00 for the identification of sepsis.

Ding and colleagues studied 96 septic patients and followed up patients for 90 days [54]. A total of 49 patients (51.0%) died at 28 days and 54 patients (56.3%) died at 90 days. When plasma samples were obtained from patients within 24 h of sepsis diagnosis and analyzed using LC-MS, the levels of plasma metabolites related to amino acid and fatty acid metabolism differed between patients who survived versus those who died. One model incorporating indoleacetic acid, 3-methylene-indolenine, heart rate, respiratory support, and the application of pressure drugs was able to predict 28-day mortality with a sensitivity of 76%, a specificity of 79%, and an AUC of 0.881. Another model incorporating dopamine, delta-12-prostaglandin J2, heart rate, respiratory support, and application of pressure drugs was able to predict 90-day mortality with a sensitivity of 83%, a specificity of 76%, and an AUC of 0.886.

When studying metabolites in septic patients, Kumar and colleagues adopted NMR spectroscopy rather than LC-MS [55]. Comparing the NMR metabolomics on admission day among 14 sepsis survivors (those who survived to day seven) and 17 sepsis non-survivors (those who succumbed on day zero), the investigators found higher serum levels of creatine, phosphocreatine, choline, betaine, tyrosine, histidine, and phenylalanine concentrations among non-survivors and hypothesized that the metabolic shutdown among non-survivors allowed for the accumulation of these metabolites. For the prediction of sepsis mortality, the higher AUCs were as follows: phosphocreatine 0.89, creatine 0.83, choline 0.76, and tyrosine 0.75.

Junru Wu and colleagues used LC-MS to perform targeted lipidomic analysis of 996 lipids on plasma samples of 193 trauma patients, who had a precise time of disease onset [56]. They then compared these results with public datasets derived from COVID-19 patients. In both trauma and COVID-19 patients, phosphatidylethanolamine elevation correlated with persistent critical illness.

Sun and colleagues compared 113 whole-blood samples and 85 serum samples from 18 severe and 15 critical COVID-19 patients (including 11 critically ill non-survivors) [57]. They acquired proteomic, metabolomic, and lipidomic data from sequencing serum samples and transcriptomic data from whole-blood cells. Differential expression analyses demonstrated differences in 2101 mRNAs, 3 proteins, 38 metabolites, and 10 lipids between the severe and critical groups. A protein–metabolite–lipid network analysis suggested that the activation of tryptophan metabolism and melatonin function disorder may explain impaired metabolism in non-survivors.

## 6. Future Directions of Omics for Critically Ill Patients

From the above examples, the clinical applications of omics are mainly for diagnosis (including metagenomic microbial identification), prognostication, and monitoring. Serial changes in biomarkers will demonstrate time-based trajectories and allow the early diagnosis of various forms of critical illness [9]. Clinicians need to be convinced that including omics within the clinical workflow improves patient outcomes compared to usual care. Omics-based biomarkers need to demonstrate some benefit when they are added to conventional clinical biomarkers. Such benefits would need to be demonstrated in adequately powered randomized trials and would need proof of generalizability via multi-center studies or replication trials.

For critical illness in general, omics studies collectively suggest the vital roles of apoptosis, systemic inflammation, and endothelial dysfunction in mediating the morbidity and mortality of various types of critical illness. Pathways that are consistently shown to be active in multiple studies should be more carefully explored for therapeutic targets. These therapeutic targets then allow the epigenetic modification or regulation of gene expression via DNA methylation, histone modification, and RNA regulation. Gene expression can be selectively turned “on” or “off” using medications that change how the body reads DNA, leading to the alterations of protein production and downstream biological effects. Unlike genome editing, an epigenetic therapeutic strategy does not change the underlying primary nucleotide sequence of DNA.

Animal studies provide several examples of omics assisting with epigenetics. Using a mouse model of ischemia–reperfusion-induced (IRI) AKI, drugs have been used to block apoptosis. Liang and colleagues found that 3-deazaneplanocin A, a selective histone methyltransferases enhancer of zest homolog-2 (EZH2), reduced the recruitment of CD3+ T cells and F4/80+ cells in kidneys; inactivated p38, resulting in the reduction of active caspase-3 and proinflammatory molecules; and ultimately decreased apoptosis [58]. Levine and colleagues found that pan-histone/protein deacetylase (HDAC) inhibition using trichostatin-enhanced miRNA 21 expression, which protected renal tubular epithelium from apoptosis [59]. Costalonga and colleagues used valproic acid, a common antiepileptic drug which also inhibits histone deacetylase and discovered both anti-apoptotic (reduced acute tubular necrosis severity and apoptotic cells count) and anti-inflammatory (reduced inflammatory cellular infiltration and the expression of proinflammatory cytokines) effects against murine IRI AKI [60].

Using a murine model of bleomycin-induced acute lung injury, Li and colleagues found that trichostatin A (the HDAC inhibitor mentioned previously) reduced lung inflammation by inhibiting the HDAC4 and serine/threonine kinase/protein kinase B pathways [61]. Kasotakis and colleagues found that trichostatin A was also able to improve the survival of mice with *Escherichia coli* pneumonia-induced acute lung injury [62]. Separately, using a mouse model of lipopolysaccharide (LPS)-induced ARDS, drugs have been used to reduce pulmonary vascular injury. Decitabine and 5-azacitidine inhibited DNA methyltransferases, reduced TNF-alpha and interleukin-1beta production and protected endothelial glycocalyx integrity [63]. Endothelial integrity in the murine LPS-induced ARDS model could also be preserved by using SU5416, a potent and selective vascular endothelial growth factor receptor (VEGFR) inhibitor, which blocked the VEGF/VEGFR and TLR4/NF-kappaB signaling pathways [64].

The epigenetic regulation of sepsis in murine models have demonstrated encouraging results. Suberoylanilide hydroxamic acid, another HDAC inhibitor, reduced production of pro-inflammatory cytokines (TNF-α, IL-1β, IL-6, and interferon gamma), and improved survival in mice [65]. Similar favorable results in mice have been obtained with other HDAC inhibitors, trichostatin A and valproic acid [66]. DNA methyltransferase inhibitors, such as procainamide and decitabine, decreased inflammation and the organ dysfunction of endotoxemic mice [67,68]. During the later phases of sepsis, when inflammation has settled, epigenetic modification may also be beneficial to reverse the post-acute-phase hypo-inflammation. Vachharajani and colleagues used a highly specific sirtuin 1 inhibitor, EX-527, in mice 24 h after the onset of sepsis. The mice demonstrated the reversal of hypo-inflammation, the restoration of leukocyte function, enhanced peritoneal bacterial clearance, and improved survival [69].

## 7. Challenges in the Clinical Application of Omics for Critically Ill Patients

For exploratory analyses and drug development, large amounts of omics data are required to illuminate pathophysiological pathways. These data may be collated and organized within integrated databases, such as a multi-omics atlas [70]. Key challenges to maintain and use a multi-omics atlas include expense, data management, data processing, and data mining. Widely available solutions include the use of data science management and artificial intelligence techniques (e.g., machine learning and deep learning). What remains challenging are artificial intelligence methods that behave like black boxes and fail to provide explanations of their predictions. This does not generate trust or confidence among clinicians or patients. Enhancements to artificial intelligence, such as explainable artificial intelligence (XAI), use methods such as feature importance ranking to help demonstrate the underlying basis of prediction models. Ultimately, omics data and the results of any artificial intelligence analyses should help clinicians make better therapeutic decisions, while clinicians should be able to feed real-world insights into databases to improve the performance of artificial intelligence methods (i.e., collaborative intelligence) [71].

To make clinical application of omics mainstream, omics technology need to be accessible and affordable for frontline clinicians. Possible solutions include the mass-processing of samples at central laboratories with rapid turnaround times, the integration of omics data with the electronic health record, chatbots to facilitate the searching of omics databases, and the translation of search results into meaningful clinical advice. In addition to the scientific and economic challenges, legal and ethical challenges include the need to ensure that any individual genetic data for individuals are obtained with informed consent, kept confidential, and shared fairly. Additionally, omics data should be representative and equitable access given across age, gender, ethnic, and socioeconomic status groups.

Finally, while animal studies provide the proof-of-concept for an epigenetic strategy, human studies are required to demonstrate clinical efficacy and safety. Blocking or modifying biological pathways may additionally require a broad approach and consideration of bypass routes. Drugs may also have unwanted side-effects, such as HDAC inhibitors increasing the risk of subsequent infection after successfully reducing inflammation. Ultimately, specific targeted therapy using existing drugs or via the creation of new pharmaceuticals, would require testing and validation through prospective clinical trials.

## 8. Conclusions

Personalized medicine, also known as precision medicine, is a medical model that acknowledges the heterogeneity of patients and differential host responses to disease. For critically ill patients with AKI, ARDS, or sepsis, omics have illuminated such heterogeneity and unveiled novel biomarkers, giving clinicians new means of diagnosis, prognosis, and monitoring. With further engineering and economic development, omics would then be more accessible and affordable for frontline clinicians. As knowledge of pathophysiological pathways mature, targeted treatments can then be developed, validated, replicated, and translated into clinical practice.

## Figures and Tables

**Table 1:** Omics for acute kidney injury.

Application	Type of Omics	Potential Clinical Applications	Author Year [Ref]
Diagnosis	Proteomics	PARK7 and CDH16 could discriminate AKI versus no AKI with the AUCs of 0.900 and 0.898. Cutoff values of PARK7 and CDH16 for identifying AKI were ≥13.557 ng/mL (sensitivity 86.7%, specificity 96.6%) and ≤53.29 pg/mL (sensitivity 83.3%, specificity 94.9%), respectively.	Li 2020 [14]
Prognosis	Genomics	SNPs in the *SERPINA4* and *SERPINA5* genes were consistently associated with AKI in two large US [15] and Finnish cohorts [15]. However, SNPs in the *BCL2* and *SIK3* genes were only found associated with AKI in the US cohort.	Frank 2012 [15] Vilander 2017 [16]
Prognosis	Genomics	One genetic variant in the CETP gene, rs1800777 (allele A), was strongly associated with lower HDL-C levels and increased risk of AKI (OR 2.38, *p* = 0.020).	Genga 2018 [18]
Prognosis	Genomics	SNPs rs41275743 and rs4648143 in the 3′-untranslated region of the nuclear factor-kappaB gene *NFKB1* raised the risk of AKI in sepsis by 1.46 and 1.56 times, respectively.	Sun 2020 [17]
Prognosis	Genomics	A short repeat in *HMOX1* was associated with AKI risk (OR 1.30 for each S-allele in an additive genetic model, 95% CI 1.01–1.66, *p* = 0.041). Alleles with a repeat number greater than 34 were significantly associated with lower heme oxygenase-1 (HO-1) concentration (*p* < 0.001).	Vilander 2019 [19]
Prognosis	Transcriptomics	Using RNA sequencing, the investigators identified and developed a 43-gene signature SVM model, which was significantly better at predicting persistent AKI than a comprehensive 58-variable clinical model (AUC 0.948, 95% CI 0.912–0.984 versus 0.739, 0.648–0.830, *p* < 0.01).	Zhang 2022 [21]

AKI: Acute kidney injury. AUC: Area under the receiver operating characteristic curve. CETP: Cholesteryl ester transfer protein. HDL-C: High-density lipoprotein cholesterol. SNP: Single nucleotide polymorphism. SVM: Support vector machine.

**Table 2:** Omics for acute respiratory distress syndrome.

Application	Type of Omics	Potential Clinical Applications	Author Year [Ref]
Diagnosis	Proteomics	Plasma malignant brain tumors 1 protein (DMBT1) was elevated in early ARDS compared to no ARDS (mean plasma concentration 2160.50 ± 94.06 vs. 1752.60 ± 111.68 pg/mL, *p* < 0.05), and was higher in severe ARDS compared to mild ARDS (mean plasma concentration 2255.26 ± 143.00 vs. 2160.50 ± 94.06 pg/mL, *p* < 0.05).	Ren 2016 [29]
Diagnosis	Metabolomics	Three breath metabolites, octane, acetaldehyde, and 3-methylheptane, were able to discriminate between ARDS and controls with an AUC of 0.80. The combination of the three-metabolite panel with the lung injury prediction score increased the AUC to 0.91.	Bos 2014 [30]
Prognosis	Transcriptomics	A higher circulating level of miRNA 887 was associated with sepsis-associated ARDS development, endothelial chemokine release, and increased neutrophil tracking.	Goodwin 2020 [31]
Prognosis	Transcriptomics	Increased miRNA 122 serum expression was an early predictor for 30-day mortality and the development of acute liver failure in patients with acute respiratory distress.	Rahmel 2018 [27]
Prognosis	Transcriptomics	Gene *ADORA3*, via the activation of various pro-inflammatory pathways, predicted prolonged time on mechanical ventilation and mortality at 28 days.	Shi 2022 [32]
Prognosis	Transcriptomics	Two miRNAs (miRNA 584 and miRNA 146a) were significantly downregulated in the serum of patients with ARDS compared to control patients.	Zhang 2021 [33]
Prognosis	Transcriptomics	Eight miRNA classifiers (namely miRNA 628.3p, miRNA 922, miRNA 505, miRNA 130b, miRNA 624, miRNA 766, miRNA 194, and miRNA 7) predicted increased mortality in moderate-to-severe ARDS.	Zhu 2016 [34]
Prognosis	Transcriptomics	miRNA 181a and miRNA 92a were risk biomarkers for ARDS, whereas miRNA 424 was a protective biomarker.	Zhu 2017 [39]
Prognosis	Proteomics	Plasma IGFBP7 moderately increased ARDS 28-day mortality (OR 1.11, 95% CI 1.04–1.19, *p* = 0.002) per log_2_ increase.	Dong 2021 [35]
Prognosis	Proteomics	Interleukin-10 concentration of 88.9 pg/mL predicted ICU mortality with a sensitivity of 73.3% and a specificity of 90.5% in severe ARDS patients receiving ECMO.	Liu 2017 [36]
Prognosis	Proteomics	A protein-based model using AGR2, NQO2, IL-1α, OSM, and TRAIL predicted in-ICU mortality. Among 23 ARDS survivors, the levels of proteins FCRL1, NTF4, and THOP1 correlated with DLCO 3 months after hospital discharge.	Molinero 2022 [28]
Prognosis	Proteomics	A 22-protein signature of low vascular protein abundance was significantly associated with lower platelet count and higher mortality in 60 ARDS patients.	Price 2022 [38]
Prognosis	Metabolomics	For predicting mortality in ARDS, the respective AUCs for phenylalanine, D-phenylalanine, and phenylacetylglutamine were 0.803, 0.785, and 0.709, respectively. The injection of phenylalanine into an ARDS mouse model increased lung injury and mortality, and the investigators hypothesized a pro-inflammatory role for phenylalanine.	Xu 2020 [37]
Treatment monitoring	Metabolomics	Serum metabolites 3-hydroxybutyrate, acetone, acetoacetate, citrate, and choline were able to predict improvement of community-acquired pneumonia and ARDS with an AUC of 0.866. Urinary metabolite 1-methylnicotinamide was able to predict the improvement of community-acquired pneumonia and ARDS with an AUC of 0.795. A combination of serum and urine metabolites was able to predict the improvement of community-acquired pneumonia and ARDS with an AUC of 0.952.	Yan 2022 [9]

ARDS: Acute respiratory distress syndrome. AUC: Area under the receiver operating characteristic curve. DLCO: Diffusing capacity for carbon monoxide (diffusing capacity of the lung). ECMO: Extracorporeal membrane oxygenation. ICU: Intensive care unit. IGFBP7: Insulin-like Growth Factor Binding Protein 7. OR: Odds ratio.

**Table 3:** Omics for sepsis.

Application	Type of Omics	Potential Clinical Applications	Author Year [Ref]
Diagnosis	Genomics	Metagenomic next-generation sequencing had higher detection rates than blood culture (88.0% versus 26.0%, *p* < 0.001) and bronchoalveolar fluid culture (92.0% versus 76.0%, *p* = 0.054).	Chien 2022 [52]
Diagnosis	Genomics Transcriptomics	For sepsis diagnosis, a plasma RNA transcriptional signature had an AUC of 0.77 for the validation set. For diagnosing a viral pathogen as the cause of sepsis, a secondary transcriptomic classifier had an AUC of 0.96 for the validation set. An integrated sepsis diagnostic model then identified 99% of microbiologically confirmed sepsis cases and predicted sepsis in 74% of suspected and 89% of indeterminate sepsis cases.	Kalantar 2022 [49]
Diagnosis	Proteomics	A total of 49 proteins, involved in the acute phase response and complement system, were associated with both survival outcome and racial background. A smaller set of 19 proteins, involved in liver cell activation, was significantly represented in patients regardless of racial/ethnic background.	Kapp 2022 [28]
Diagnosis	Proteomics	An index combining the levels of four proteins (β2-microglobulin >3.7 mg/L, VCAM1 > 2216.8 ng/mL, ApoC3 ≤ 54.532 μg/mL, ApoE > 62.45 mg/L) and two conventional infection biomarkers (procalcitonin, C-reactive protein yielded an AUC of 0.772 for sepsis identification.	Li 2022 [51]
Diagnosis	Proteomics	A three-protein signature (S100A8, lactotransferrin, actinin 1) discriminated patients with and without ventilator-associated pneumonia, with an optimal sensitivity-specificity profile of 93% and 94%, respectively.	Nguyen 2013 [46]
Diagnosis	Metabolomics	A regression model incorporating 10 amino acid and lipid metabolites (assayed from plasma and urine) and five traditional physiological indicators had a perfect AUC of 1.00 for the identification of sepsis	Su 2022 [53]
Prognosis	Genomics	A variant of the *FER* (Fps/Fes related tyrosine kinase) gene called rs4957796 was strongly associated with improved 28-day survival in patients with sepsis and pneumonia (HR for mortality 0.56, 95% CI 0.45–0.69).	Rautanen 2015 [7]
Prognosis	Transcriptomics	Decreased levels of two microRNAs (miRNA 103 and miRNA 107) predicted the risk of concomitant ARDS and 28-day mortality.	Wang 2020 [47]
Prognosis	Proteomics	A proteomic signature of microvascular dysfunction predicted the composite endpoint of 28-day mortality and/or intubation with an AUC of 0.90 (95% CI 0.86–0.94, *p* < 0.0001).	Rovas 2022 [50]
Prognosis	Proteomics	Nine proteins (GPX3, APOB, ORM1, SERPINF1, LYZ, C8A, CD14, APOC3, and C1QC) were associated with organ dysfunction (defined by SOFA score > 6) with a sensitivity of 81%, a specificity of 84%, and an of AUC 0.82. In addition, 22 proteins (CLU, LUM, APOL1, SAA1, CLEBC3B, C8A, ITIH4, KNG1, AGT, C7, SAA2, APOH, HRG, AFM, APOE, APOC1, C1S, SERPINC1, IGFALS, KLKB1, CFB, and BTD) were associated with mortality with a sensitivity of 91%, a specificity of 72%, and an AUC of 0.81.	Ruiz-Sanmartín 2022 [48]
Prognosis	Metabolomics	One model incorporating indoleacetic acid, 3-methylene-indolenine, heart rate, respiratory support, and the application of pressure drugs could predict 28-day mortality with a sensitivity of 76%, a specificity of 79%, and an AUC of 0.881. Another model incorporating dopamine, delta-12-prostaglandin J2, heart rate, respiratory support, and the application of pressure drugs was able to predict 90-day mortality with a sensitivity of 83%, a specificity of 76%, and an AUC of 0.886.	Ding 2022 [54]
Prognosis	Metabolomics	Higher serum levels of creatine, phosphocreatine, choline, betaine, tyrosine, histidine, and phenylalanine concentrations were found among non-survivors, leading to the hypothesis that the metabolic shutdown among non-survivors allowed for accumulation of these metabolites. For the prediction of sepsis mortality, the higher AUCs were as follows: phosphocreatine 0.89, creatine 0.83, choline 0.76, and tyrosine 0.75.	Kumar 2022 [55]
Prognosis	Metabolomics	In common between trauma and COVID-19 patients, phosphatidylethanolamine elevation correlated with persistent critical illness.	Wu 2022 [56]
Prognosis	Genomics Transcriptomics Proteomics Metabolomics	A trans-omics approach encompassing genomic, transcriptomic, proteomic, and metabolomic profiles revealed neutrophil heterogeneity between asymptomatic and critically ill patients. In critically ill COVID-19 patients, neutrophil over-activation, arginine depletion, and tryptophan metabolites accumulation were present, which correlated with T cell dysfunction.	Wu 2021 [49]
Prognosis	Transcriptomics Proteomics Metabolomics	A total of 2101 mRNAs, 3 proteins, 38 metabolites, and 10 lipids were differentially expressed between severe and critically ill COVID-19 patients.	Sun 2021 [57]

ARDS: Acute respiratory distress syndrome. AUC: Area under the receiver operating characteristic curve. HR: Hazard ratio. SOFA: Sequential Organ Failure Assessment.

## Data Availability

All data used can be found in the text and tables.
